# Extended-Spectrum β-Lactamase–producing *Escherichia coli* in Neonatal Care Unit

**DOI:** 10.3201/eid1706.101868

**Published:** 2011-06

**Authors:** James R. Johnson

**Affiliations:** Author affilation: Veterans Affairs Medical Center, Minneapolis, Minnesota, USA

**Keywords:** Escherichia coli, bacteria, outbreak, colonization, extended-spectrum β-lactamase, neonatal care unit, urinary tract infection, antimicrobial resistance, letter

**To the Editor:** Tschudin-Sutter et al. provide convincing evidence of transfer of an extended-spectrum β-lactamase–producing *Escherichia coli* strain from a mother to her vaginally delivered twins, then from the neonates to a health care worker and other neonates in a neonatal care unit (*1*). This finding advances our understanding of how extended-spectrum β-lactamase–positive (and, by extension, other antimicrobial drug–resistant or virulent strains) *E*. *coli* can spread within the community.

However, the authors’ use of the term infection for the asymptomatic colonization that was observed, including in the mother (who had asymptomatic bacteriuria), is potentially misleading. This term could perpetuate a line of thinking that is all too common among clinicians and leads to unnecessary antimicrobial drug use, thereby ironically aggravating the problem of antimicrobial drug resistance.

Although the first paragraph of their report implicitly acknowledges the distinction between infection and colonization, the rest of the report (including the abstract) uses the terms infection or infected interchangeably with colonization or colonized. Examples include “Subsequently, infection spread by healthcare worker contact with other neonates,” “a healthcare worker also was infected,” and “a urinary tract infection developed....”

One wonders why, in the absence of genitourinary symptoms, the (postpartum) mother’s urine was cultured and why the positive culture prompted antimicrobial drug therapy. This seeming misinterpretation by the mother’s providers of what probably was a harmless colonization state as representing acute disease, and their all too typical response (i.e., antimicrobial drug therapy), are to be discouraged (*2*). More cautious use of terminology, to emphasize the distinction between colonization and infection (which have radically different therapeutic implications), may help refine clinicians’ thinking and practice in this regard, thereby promoting improved antimicrobial drug stewardship and slowing the antimicrobial drug resistance epidemic.
