# Supplementary material for: Extended-Spectrum β-Lactamase–producing Escherichia coli in Neonatal Care Unit
Source: Emerg Infect Dis. 2011 Jun;17(6):1153–4. doi: 10.3201/eid1706.101868 (PMC3321532; doi:10.3201/eid1706.101868)
Supplement: Related texts

# Reply: Extended-Spectrum β-Lactamase–producing *Escherichia coli* in Neonatal Care Unit

**Authors:** Sarah Tschudin-Sutter, Reno Frei, Manuel Battegay, Irene Hoesli, Andreas F. Widmer

**Affiliations:** Author affiliations: University Hospital Basel, Switzerland

**In Response:** We thank James Johnson for the issue that he has raised in his letter (*1*). We agree that distinction of the terms colonization and infection is crucial to prevent misinterpretation of clinical findings and subsequently unnecessary antimicrobial drug use. The outbreak occurred in the hospital; therefore, definitions of nosocomial infections were used throughout the article (*2*).

Nosocomial urinary tract infection is defined by the Centers for Disease Control and Prevention as asymptomatic bacteriuria or symptomatic infection (urinary tract infection–symptomatic urinary tract infection; www.cdc.gov/nhsn/PDFs/pscManual/17pscNosInfDef_current.pdf) (*3**,**4*). Therefore, the term nosocomial urinary tract infection in our report is correct. However, we agree with the author that the term asymptomatic bacteriuria is less than optimal and it was removed when we submitted our report. We agree that the term infection is misleading for describing spread to health care workers and that colonization should have been used. However, the article clearly states that invasive infection did not occur in any of the neonates or health care workers found to be colonized. In addition, the focus of the article was to describe the mode of transmission rather than the distinction between colonization and infection.

## References

[1] Johnson JR. Extended-spectrum β-lactamase–producing *Escherichia coli* in neonatal care unit. Emerg Infect Dis. 2011;17:1153–4. 10.3201/eid1706.10186821749754PMC3321532

[2] Tschudin-Sutter S, Frei R, Battegay M, Hoesli I, Widmer AF. Extended-spectrum β-lactamase–producing *Escherichia coli* in neonatal care unit. Emerg Infect Dis. 2010;16:1758–60.2102953710.3201/eid1611.100366PMC3294509

[3] Garner JS, Jarvis WR, Emori TG, Horan TC, Hughes JM. CDC definitions for nosocomial infections, 1988. Am J Infect Control. 1988;16:128–40. 10.1016/0196-6553(88)90053-32841893

[4] Horan TC, Andrus M, Dudeck MA. CDC/NHSN surveillance definition of health care-associated infection and criteria for specific types of infections in the acute care setting. Am J Infect Control. 2008;36:309–32. 10.1016/j.ajic.2008.03.00218538699
